# Vertebral Artery Hypoplasia and Posterior Circulation Vulnerability: A Systematic Review of Epidemiological, Hemodynamic, and Clinical Evidence

**DOI:** 10.3390/neurolint18090169

**Published:** 2026-09-01

**Authors:** Olja Mirkovic, Milos Stepovic, Jovana Milosavljevic, Maja Vulovic, Ivana Zivanovic-Macuzic, Dejan Aleksic, Milos N. Milosavljevic, Melanija Tepavcevic, Ivona Marinkovic, Simonida Delic, Kristijan Jovanovic, Verica Vukicevic, Ana Tomic, Miljana Maric, Jelena Kostic

**Affiliations:** 1 Faculty of Medical Sciences, University of Kragujevac, 34000 Kragujevac, Serbia; oljam151@gmail.com (O.M.); verica.vukicevic@gmail.com (V.V.); 2 Department of Anatomy, Faculty of Medical Sciences, University of Kragujevac, 34000 Kragujevac, Serbia; maja@fmn.kg.ac.rs (M.V.); ivanaanatom@yahoo.com (I.Z.-M.); melanijatepavcevic@yahoo.com (M.T.); ivbankovic1@gmail.com (I.M.); simonida.delic@fmn.kg.ac.rs (S.D.); kralj100@yahoo.com (K.J.); 3 Department of Neurology, Faculty of Medical Sciences, University of Kragujevac, 34000 Kragujevac, Serbia; drdeal1987@gmail.com; 4 Department of Pharmacology and Toxicology, Faculty of Medical Sciences, University of Kragujevac, 34000 Kragujevac, Serbia; milosavljevicmilos91@gmail.com; 5 Emergency Medical Service, Institute for Emergency Medical Services, 11000 Belgrade, Serbia; 6 Clinical Center of Serbia, Center for Radiology, 11000 Belgrade, Serbia; anatomic9977@gmail.com (A.T.); miljanamaric4@gmail.com (M.M.); jecko6yu@yahoo.com (J.K.); 7 Medical Faculty, University of Belgrade, 11000 Belgrade, Serbia

**Keywords:** clinical anatomy, vertebral artery hypoplasia, posterior circulation stroke, vertebrobasilar insufficiency, cerebrovascular hemodynamics, vascular anatomical variation

## Abstract

Background/Objectives: Vertebral artery hypoplasia is a common congenital anatomical variant of the vertebral arteries, traditionally considered a benign finding. However, accumulating evidence suggests that VAH may influence vertebrobasilar hemodynamics and contribute to susceptibility to posterior circulation disorders. This systematic review aimed to synthesize current evidence regarding the epidemiology, hemodynamic significance, neurological manifestations, and cerebrovascular outcomes associated with VAH. Methods: This systematic review was conducted according to the PRISMA 2020 guidelines and registered in PROSPERO (CRD420261442242). A systematic search of PubMed/MEDLINE was performed from database inception to 8 June 2026, supplemented by manual reference screening and targeted searches. Studies evaluating VAH diagnosed using vascular imaging or anatomical assessment, including CTA, MRA, Doppler ultrasonography, and DSA, were eligible. Methodological quality was assessed using Joanna Briggs Institute critical appraisal tools, and the level of evidence was classified according to the Oxford Centre for Evidence-Based Medicine framework. Results: Forty-six studies were included in the qualitative synthesis. VAH prevalence varied substantially according to imaging modality, diagnostic criteria, and study population, ranging from approximately 3% to nearly 50%. Across clinical cohorts, VAH was frequently associated with posterior circulation ischemic stroke, transient ischemic attack, and vertebrobasilar insufficiency. Hemodynamic studies demonstrated reduced vertebral artery flow, increased vascular resistance, impaired cerebrovascular reactivity, and altered collateral flow redistribution. VAH was also associated with vestibular disorders, non-stroke neurological syndromes, vertebral artery dissection, and other cerebrovascular anatomical variations. Unlike previous reviews focusing primarily on prevalence or stroke associations, this review integrates epidemiological, anatomical, hemodynamic, computational, and neurological evidence within a unified clinically oriented framework and proposes an evidence-informed conceptual model of VAH as a context-dependent low-flow vascular susceptibility phenotype. Conclusions: Current evidence indicates that VAH represents a clinically relevant vascular susceptibility phenotype rather than merely an incidental anatomical variant. Its clinical significance appears to depend on the interaction between reduced vertebral artery flows, collateral capacity, vascular remodeling, and acquired cerebrovascular risk factors. Standardized diagnostic criteria and prospective multimodal studies are required to define its role in future cerebrovascular risk assessment.

## 1. Introduction

The vertebrobasilar arterial system supplies the brainstem, cerebellum, thalamus, occipital lobes, and portions of the temporal lobes, making it essential for numerous neurological functions [1]. Although posterior circulation accounts for only approximately 20–25% of all cerebral blood flow, ischemia within this vascular territory is associated with substantial morbidity and mortality because of the critical structures involved [2]. Anatomical variations of the vertebrobasilar circulation are common and may influence cerebral perfusion, collateral circulation, and susceptibility to ischemic events [3,4].

Vertebral artery hypoplasia (VAH) is one of the most common congenital variations of the vertebral arteries and is characterized by underdevelopment of one vertebral artery [5]. Despite its frequent identification during vascular imaging, no universally accepted diagnostic definition exists. Previous studies have defined VAH using arterial diameter thresholds ranging from 2.0 to 3.0 mm, side-to-side diameter asymmetry, or flow-based ultrasonographic criteria, resulting in considerable heterogeneity across the literature [6,7,8]. Reported prevalence varies widely according to imaging modality, diagnostic criteria, and study population, ranging from approximately 3% in population-based Doppler studies to nearly 50% in selected cerebrovascular cohorts [9,10].

For many years, VAH was considered a benign congenital anatomical variant because compensatory enlargement of the contralateral vertebral artery was thought to preserve adequate vertebrobasilar blood flow [11]. Indeed, physiological and ultrasonographic studies have demonstrated compensatory increases in contralateral vertebral artery flow in many individuals with unilateral VAH [6,12]. However, accumulating evidence suggests that this compensation may be insufficient under pathological conditions. Several observational studies have demonstrated that VAH is significantly associated with posterior circulation ischemic stroke, transient ischemic attack, vertebrobasilar insufficiency, and other disorders involving the posterior cerebral circulation [12,13,14]. Moreover, studies have identified VAH as an independent statistical association of posterior circulation ischemia after adjustment for conventional vascular risk factors, suggesting that congenital vascular anatomy may contribute to cerebrovascular vulnerability beyond established risk factors [11,15,16,17].

In addition to ischemic stroke, VAH has been implicated in a broader spectrum of neurological disorders, including isolated vertigo, vestibular syndromes, transient global amnesia, vertebrobasilar insufficiency, and other manifestations potentially related to impaired posterior circulation perfusion [13,14,15,17]. Proposed pathophysiological mechanisms include reduced vertebral artery flow volume, impaired cerebrovascular autoregulation, increased vascular resistance, asymmetric vertebrobasilar perfusion, and altered local hemodynamics, all of which may reduce perfusion reserve and increase susceptibility to ischemic injury [18,19,20,21].

Despite the growing body of evidence, the clinical significance of VAH remains controversial. Published studies differ substantially with respect to patient populations, imaging modalities, diagnostic thresholds, outcome definitions, and methodological quality, resulting in inconsistent conclusions regarding whether VAH represents merely a benign anatomical variant or a clinically relevant cerebrovascular risk factor [22]. Furthermore, available evidence is dispersed across neuroradiological, neurological, vascular, and hemodynamic research, limiting comprehensive interpretation of its clinical implications.

To our knowledge, no previous systematic review has comprehensively synthesized the available evidence domains within a single clinically oriented framework while simultaneously evaluating the methodological quality of the included studies. Addressing this knowledge gap may improve understanding of the role of VAH in posterior circulation disorders and help clinicians interpret this common vascular variant in everyday practice.

Therefore, the aim of this systematic review was to comprehensively evaluate the current evidence regarding the clinical significance of vertebral artery hypoplasia, with particular emphasis on its association with neurological manifestations and cerebrovascular outcomes, including posterior circulation stroke, transient ischemic attack, vertebrobasilar insufficiency, hemodynamic alterations, and related ischemic events.

Specifically, this review integrates evidence regarding: epidemiology and prevalence; diagnostic criteria and imaging definitions; cerebral hemodynamics and flow measurements; collateral circulation and Circle of Willis anatomy; computational fluid dynamics; vascular remodeling; posterior circulation ischemic stroke; non-stroke neurological syndromes; anatomical variants associated with VAH.

### Novelty and Contribution to the Current Literature

Previous systematic reviews and meta-analyses have primarily addressed specific aspects of vertebral artery hypoplasia, particularly its anatomical prevalence and diagnostic characteristics or its association with posterior circulation ischemia. To our knowledge, no previous review has jointly synthesized epidemiological, anatomical, quantitative hemodynamic, collateral, vascular remodeling, computational fluid dynamics, and neurological evidence within a single clinically oriented framework. The present review additionally integrates evidence across distinct clinical and biological phenotypes, including asymptomatic and healthy populations, posterior circulation stroke, transient ischemic attack and vertebrobasilar insufficiency, vestibular and other non-stroke neurological manifestations, and vascular complications such as vertebral artery dissection. In contrast, recent systematic reviews focusing on the anatomical prevalence and diagnostic characteristics of VAH have primarily addressed the frequency and definition of the variant, without integrating these findings with quantitative hemodynamic, collateral, vascular remodeling, computational, and non-stroke neurological evidence. The formal use of Joanna Briggs Institute critical appraisal tools together with Oxford Centre for Evidence-Based Medicine evidence-level classification further provides a structured assessment of methodological quality and evidence strength across these heterogeneous domains. Rather than establishing a causal relationship between VAH and cerebrovascular disease, the present synthesis provides an evidence-informed conceptual framework in which VAH is interpreted as a context-dependent low-flow vascular susceptibility phenotype whose clinical expression may depend on collateral capacity, coexisting vascular abnormalities, vascular remodeling, and acquired cerebrovascular risk factors.

## 2. Materials and Methods

### 2.1. Study Design

This systematic review was conducted and reported in accordance with the Preferred Reporting Items for Systematic Reviews and Meta-Analyses (PRISMA 2020) guidelines [23]. The review was designed to evaluate the clinical significance of VAH and its association with neurological and cerebrovascular outcomes reported in the available literature. It was registered with PROSPERO ID: CRD420261442242.

The review protocol was developed before study selection and followed a predefined methodology that included a structured literature search, eligibility assessment, data extraction, methodological quality assessment, and qualitative evidence synthesis. Due to substantial heterogeneity among the included studies regarding study design, imaging modalities, definitions of vertebral artery hypoplasia, and reported clinical outcomes, a qualitative systematic review was performed without quantitative meta-analysis.

### 2.2. Research Question

The review was designed according to the Population–Exposure–Comparison–Outcome (PECO) framework [24].

Population (P): Adults undergoing imaging evaluation supplemented by anatomical studies of human specimens of the vertebral arteries using computed tomography angiography (CTA), magnetic resonance angiography (MRA), Doppler ultrasonography, or digital subtraction angiography (DSA).

Exposure (E): Presence of VAH, defined according to study-specific diagnostic criteria, most commonly based on vertebral artery diameter thresholds or side-to-side asymmetry demonstrated by vascular imaging.

Comparison (C): Individuals without VAH or with normal-caliber vertebral arteries, when a comparison group was available. In descriptive anatomical studies and case reports, the reference comparison was normal vertebral artery anatomy.

Outcome (O): The primary outcomes included posterior circulation ischemic stroke, transient ischemic attack (TIA), vertebrobasilar insufficiency, vertebral artery stenosis, and other neurological manifestations associated with posterior circulation ischemia. Secondary outcomes included the prevalence of vertebral artery hypoplasia, imaging characteristics, vascular anatomical variations, laterality, and associations with other cerebrovascular risk factors.

The research question was formulated as follows: “What is the clinical significance of vertebral artery hypoplasia in relation to neurological and cerebrovascular outcomes?”

### 2.3. Eligibility Criteria

Studies were selected according to predefined eligibility criteria established before the literature search.

#### 2.3.1. Inclusion Criteria

Studies were considered eligible if they were original research articles involving human participants and employed observational study designs, including prospective or retrospective cohort studies, case–control studies, cross-sectional studies, case series, or case reports as well as controlled human physiological studies evaluating vertebral artery hemodynamics or vascular reactivity. Eligible studies evaluated VAH as a distinct anatomical or vascular variant, with the diagnosis established using validated imaging modalities such as computed tomography angiography (CTA), magnetic resonance angiography (MRA), Doppler ultrasonography, color-coded duplex ultrasonography, digital subtraction angiography (DSA), or anatomical dissection in cadaveric studies. Additionally, studies were required to report at least one clinically relevant neurological, cerebrovascular, anatomical, or hemodynamic outcome related to VAH, be published in English, and have the full text available for review. Computational and mechanistic studies based on human imaging data were also eligible when they evaluated VAH-related hemodynamic mechanisms.

Studies were considered eligible if they investigated at least one clinically relevant outcome related to vertebral artery hypoplasia (VAH), including posterior circulation ischemic stroke, vertebrobasilar ischemic stroke, transient ischemic attack (TIA), vertebrobasilar insufficiency, vertebral artery stenosis or other forms of vascular compromise, vestibular disorders associated with the vertebrobasilar circulation, anatomical characteristics and prevalence of VAH, imaging features of VAH, or its hemodynamic consequences and effects on collateral cerebral circulation.

#### 2.3.2. Exclusion Criteria

Studies were excluded if they were animal or experimental studies, in vitro investigations, review articles, systematic reviews, meta-analyses, editorials, letters to the editor, expert opinions, conference abstracts, or book chapters. Additional exclusion criteria included studies that did not specifically evaluate VAH as a distinct anatomical entity, lacked relevant neurological, cerebrovascular, anatomical, imaging, or hemodynamic outcomes, were published in languages other than English, or did not have an accessible full-text version.

Studies describing rare anatomical variants or individual clinical cases were retained when they provided unique evidence regarding the anatomical configuration, imaging characteristics, hemodynamic significance, or clinical implications of vertebral artery hypoplasia.

### 2.4. Information Sources

A systematic literature search was conducted in the PubMed/MEDLINE electronic database from database inception to 8 June 2026. PubMed/MEDLINE was selected as the primary database because it provides comprehensive coverage of peer-reviewed biomedical literature and indexes the majority of studies related to vascular anatomy, neurovascular imaging, and cerebrovascular diseases. The Cochrane Library, Wiley Online Library, and, additionally, Google Scholar were searched.

To ensure comprehensive identification of all relevant publications, supplementary manual searches were performed by screening the reference lists of all included articles and relevant review papers. In addition, targeted searches were undertaken using combinations of VAH with specific neurological and cerebrovascular outcomes to identify potentially eligible studies that might not have been retrieved by the primary search strategy. No restrictions regarding publication date were applied.

### 2.5. Search Strategy

A comprehensive search strategy was developed a priori using a combination of Medical Subject Headings (MeSH) and free-text terms related to vertebral artery hypoplasia and cerebrovascular disease, combined with the Boolean operators AND and OR. The primary search strategy was (“vertebral artery hypoplasia” OR “hypoplastic vertebral artery” OR VAH) AND (“posterior circulation stroke” OR stroke OR ischemic stroke OR ischemia OR TIA OR “transient ischemic attack” OR “vertebrobasilar insufficiency”), which identified 82 potentially eligible records. To maximize retrieval, additional targeted searches were subsequently performed using broader search combinations, including vertebral artery hypoplasia with stroke (59 records), posterior circulation stroke (14 records), ischemia (39 records), and vertebrobasilar insufficiency (32 records). The Cochrane Library and Wiley Online Library were additionally searched using combinations of terms related to vertebral artery hypoplasia, vertebral artery, posterior circulation, stroke, ischemia, and vertebrobasilar insufficiency. These searches identified 4 records in the Cochrane Library and 44 records in Wiley Online Library. All retrieved records were combined into a single database, and duplicate records were removed before the screening process. Searches were limited to studies involving human participants and publications in English, with no restriction on publication year to ensure comprehensive identification of all relevant studies from database inception. Title and abstract screening, full-text eligibility assessment, study selection, and data extraction were performed independently by two reviewers, with disagreements resolved through discussion and, when necessary, consultation with a third reviewer.

### 2.6. Study Selection Process

**Figure 1:**
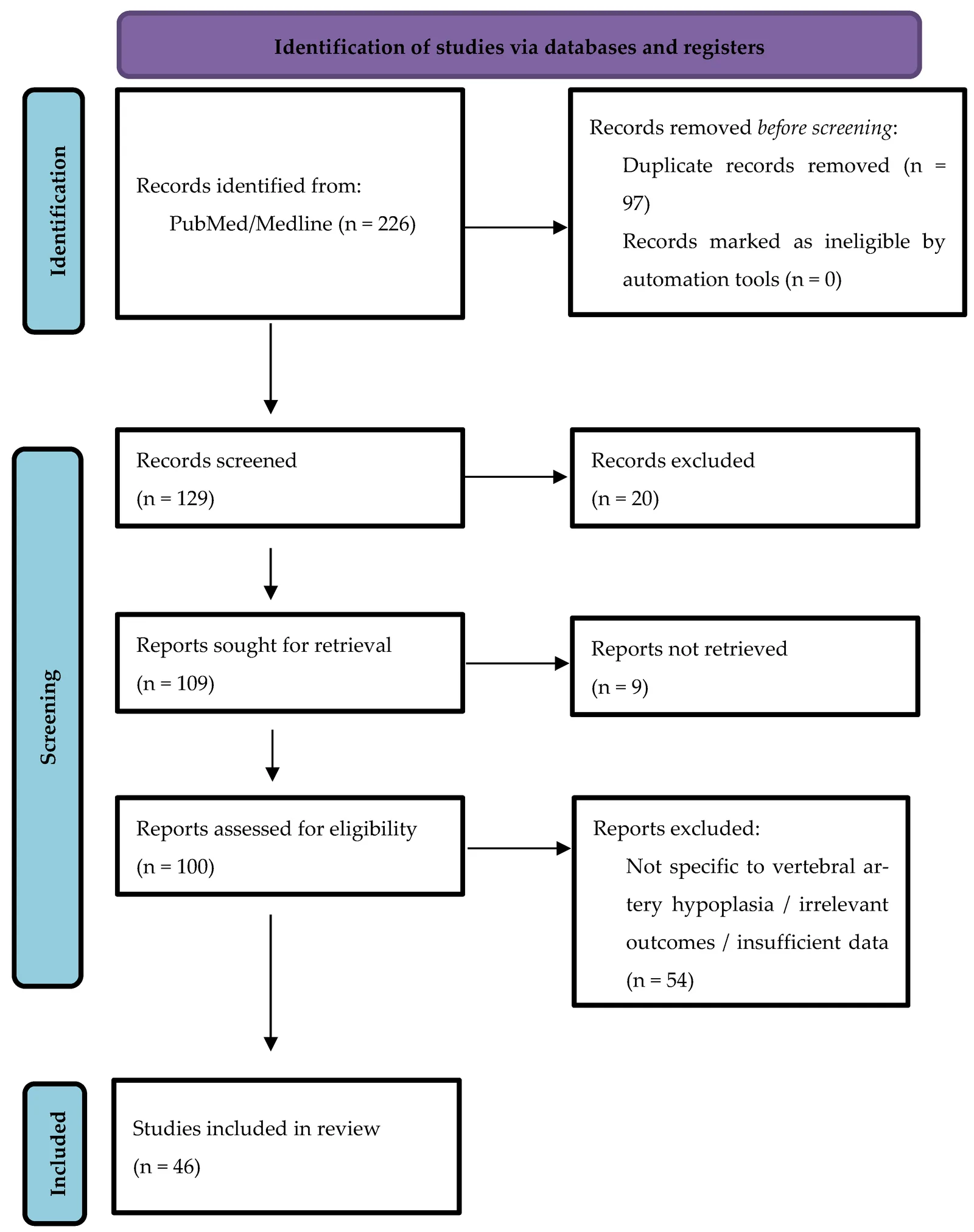
PRISMA 2020 flow diagram illustrating the study selection process based on the primary PubMed/MEDLINE search; supplementary searches of Cochrane Library, Wiley Online Library, Google Scholar, and reference lists were conducted to identify additional eligible records.

Records identified through the database and supplementary searches were screened according to the predefined eligibility criteria after duplicate removal. Potentially eligible articles underwent full-text assessment, and studies not meeting the inclusion criteria were excluded. A total of 46 studies were included in the final qualitative synthesis. The complete study selection process is presented in the PRISMA 2020 flow diagram (Figure 1).

### 2.7. Data Collection Process

Data extraction was performed using a standardized data extraction form that was developed before the review commenced. Each eligible study was reviewed in detail, and all relevant information was extracted in a consistent manner to ensure comparability across studies.

For each included study, data were extracted on the first author, year of publication, country of origin, study design, study population, sample size, participant characteristics, imaging modality used to diagnose VAH, diagnostic criteria for VAH, comparison group (when applicable), primary and secondary neurological or cerebrovascular outcomes, principal findings, methodological quality (risk of bias), and level of evidence according to the Oxford Centre for Evidence-Based Medicine (OCEBM).

When multiple publications reported overlapping study populations, only the most comprehensive publication or the study with the largest dataset was included in the qualitative synthesis to avoid duplication of data. Where relevant information was missing or incompletely reported; only the data explicitly presented in the published article were extracted, and unavailable variables were recorded as not reported.

In addition, data required for evidence synthesis were extracted according to the PECO framework (Population, Exposure, Comparison, and Outcomes), enabling standardized comparison across studies despite methodological heterogeneity.

### 2.8. Data Items

The primary outcomes of interest were neurological and cerebrovascular manifestations potentially associated with VAH. These included posterior circulation ischemic stroke, vertebrobasilar ischemia, transient ischemic attack (TIA), vertebrobasilar insufficiency, recurrent ischemic events, and other clinically relevant neurological disorders involving the posterior cerebral circulation.

Secondary outcomes included the prevalence of vertebral artery hypoplasia, vertebral artery diameter, side predominance (right or left VAH), vascular asymmetry, imaging characteristics, associated vascular anomalies, vertebral artery stenosis, collateral circulation, hemodynamic parameters, and anatomical variations involving the vertebral and posterior circulation.

### 2.9. Risk of Bias Assessment

The methodological quality and risk of bias of the included studies were assessed using the Joanna Briggs Institute (JBI) Critical Appraisal Checklists [25]. Design-specific JBI tools were applied according to the methodological design of each study (cohort, case–control, analytical cross-sectional, case series, or case report). The assessment evaluated key methodological domains, including participant selection, exposure measurement, outcome assessment, control of potential confounding, completeness of outcome reporting, and, where applicable, the appropriateness of the statistical analysis.

For each study, the overall methodological quality was categorized as low, moderate, or high risk of bias based on the proportion of fulfilled JBI criteria. Studies fulfilling most appraisal criteria with minimal methodological limitations were considered to have a low risk of bias, whereas studies with important methodological shortcomings or inherent limitations related to study design were classified as having a moderate or high risk of bias. Risk of bias assessment was incorporated into the qualitative interpretation of the findings and was considered during evidence synthesis.

### 2.10. Level of Evidence Assessment

In addition to the methodological quality assessment, the level of evidence of each included study was classified according, where applicable, to the Oxford Centre for Evidence-Based Medicine (OCEBM) Levels of Evidence [26]. The OCEBM framework was selected because it provides a standardized and widely accepted hierarchy for grading clinical evidence based on study design and methodological characteristics.

For the purposes of the present review, evidence levels were operationalized according to the methodological designs represented among the included studies. Prospective cohort studies were classified as Level II, retrospective cohort and case–control studies as Level III, cross-sectional studies and case series as Level IV, and case reports as Level V. Controlled human physiological studies were also classified as Level III, reflecting their experimental design and direct assessment of physiological responses. Computational/mechanistic studies based on human imaging data were classified separately and were not assigned an OCEBM level because the OCEBM framework does not provide a specific evidence level for computational modeling studies.

The level of evidence was determined independently of the risk of bias assessment. While the JBI Critical Appraisal Checklists were used to evaluate the methodological quality of individual studies, the OCEBM classification was applied to indicate the relative strength of evidence provided by each study design. Thus, a study could have a low risk of bias while still being classified as a lower level of evidence because of its observational study design.

The assigned levels of evidence are presented in the summary table of included studies and were considered during interpretation of the overall findings.

### 2.11. Effect Measures

As this review did not include a quantitative meta-analysis, no pooled effect estimates were calculated. Instead, the effect measures reported by the individual studies were extracted and summarized qualitatively.

For anatomical and imaging studies that did not report comparative effect measures, descriptive outcomes such as vertebral artery diameter, prevalence of vertebral artery hypoplasia, anatomical variations, vascular asymmetry, and imaging characteristics were collected and synthesized qualitatively.

### 2.12. Data Synthesis

A quantitative meta-analysis was not performed because of substantial methodological and clinical heterogeneity among the included studies. The studies differed considerably with respect to study design, study populations, sample size, imaging modalities used for the diagnosis of vertebral artery hypoplasia, diagnostic definitions of VAH, comparator groups, and reported neurological outcomes. These differences precluded meaningful statistical pooling of the available data.

Therefore, a qualitative synthesis was undertaken. Following data extraction, studies were systematically grouped according to their primary research focus to facilitate comparison of findings across heterogeneous study designs. The main synthesis categories included:

1. Studies evaluating the association between VAH and posterior circulation ischemic stroke
2. Studies investigating transient ischemic attack and vertebrobasilar insufficiency and association with VAH
3. Studies examining vestibular and other neurological manifestations associated with VAH
4. Imaging and anatomical studies evaluating the prevalence, morphology, and diagnostic characteristics of VAH
5. Studies describing rare anatomical variants and clinically relevant case reports of VAH

Within each synthesis category, findings were summarized according to study design, study population, diagnostic criteria for vertebral artery hypoplasia, principal outcomes, methodological quality, and level of evidence. Particular attention was given to similarities and differences in study methodology, diagnostic thresholds for VAH, imaging techniques, and reported clinical associations.

The findings of the included studies were summarized in structured evidence tables presenting study characteristics, methodological quality, risk of bias assessment, level of evidence, and principal outcomes. This approach enabled direct comparison of studies despite methodological heterogeneity and facilitated interpretation of the overall body of evidence.

## 3. Results

Given the substantial heterogeneity in VAH definitions, imaging modalities, populations, comparators, outcome definitions, and reported effect measures, no quantitative meta-analysis was performed and therefore no pooled estimates of association or formal heterogeneity statistics were calculated.

Across all included studies, the publication years ranged from 1999 to 2026, reflecting a broad temporal span of evidence on vertebral artery hypoplasia and its anatomical, hemodynamic, and clinical associations [27,28,29,30,31,32,33,34,35,36,37,38,39,40,41,42,43,44,45,46,47,48,49,50,51,52,53,54,55,56,57,58,59,60,61,62,63,64,65,66,67,68,69,70,71,72].

### 3.1. Prevalence and Epidemiology of VAH

The included studies (Supplementary Table S1) comprised cerebrovascular, symptomatic, and healthy populations, with VAH assessed via CTA, MRA, or ultrasound using variable diagnostic criteria [27,28,29,30,31,32,33,34,35]. Comparators included non-VAH subjects or alternative vascular conditions, while outcomes covered stroke distribution, hemodynamic parameters, and anatomical variability.

Overall methodological quality (Supplementary Table S2) ranged from low to moderate risk of bias. Most studies had appropriate imaging and clearly defined populations, but confounding was often insufficiently addressed, particularly in retrospective designs [27,28,29,30,31,32,33,34,35].

VAH showed marked variability in prevalence across study designs, populations, and imaging modalities, ranging from approximately 3% to nearly 50%. In large imaging-based cohorts, Fortuniak et al. [27] reported VAH in 20% of individuals undergoing CTA, while Min & Lee [30] found a similar prevalence of 23.9% in healthy adults, supporting VAH as a common anatomical variant. Higher prevalence rates were observed in symptomatic MRI populations, where Peterson et al. [28] reported VAH in 43.5% of patients with neck pain, and Çiçek et al. [29] found a prevalence of 32.4% in patients with headache, neck pain, and dizziness, without a significant association with arcuate foramen variation.

Ultrasound-based studies showed lower but functionally defined prevalence. Chen et al. reported VAH-related flow abnormalities in 3.3–5.9% of individuals depending on diagnostic criteria, while a complementary hemodynamic analysis identified low-flow VAH in approximately 4.3%, emphasizing the importance of functional rather than purely morphological definitions [31].

In cerebrovascular populations, higher prevalence was consistently reported. Siva et al. [32] observed VAH in 47.0% of patients undergoing CTA for cerebrovascular evaluation, while Wang et al. [33] identified VAH-related hemodynamic abnormalities in symptomatic patients with dizziness and headache. Vilimas et al. [34] demonstrated a significantly higher frequency of smaller non-dominant vertebral arteries in posterior circulation stroke compared with controls.

In vertebrobasilar ischemia–related conditions, Li et al. [35] reported a VAH prevalence of 40.6% in patients with vertebrobasilar transient ischemic attacks, further supporting its association with posterior circulation pathology and multivessel disease. It can be concluded that VAH represents a relatively frequent anatomical variant, with prevalence strongly influenced by imaging modality, diagnostic thresholds, and patient selection, with higher prevalence frequently reported in symptomatic and cerebrovascular populations (Table 1).

### 3.2. Association Between Vertebral Artery Hypoplasia and the Risk of Posterior Circulation Stroke

The included studies (Supplementary Table S3) investigated the association between VAH and posterior circulation ischemia across stroke, transient ischemic attack, vertigo, neurological, and control populations. VAH was assessed by MRI, MRA, CTA, ultrasound, or DSA using variable diameter, asymmetry, or flow-based criteria, and outcomes included posterior circulation infarction, vertebrobasilar ischemia, and related hemodynamic and anatomical findings [36,37,38,39,40,41,42,43,44,45,46,47,48,49,50,51,52,53].

Overall methodological quality (Supplementary Table S4) was generally high, with most studies showing low to moderate risk of bias on the JBI checklist. Imaging and outcome assessment were well standardized, although residual confounding, retrospective single-center designs, and heterogeneous VAH definitions remained the main limitations [36,37,38,39,40,41,42,43,44,45,46,47,48,49,50,51,52,53].

Across the included studies, VAH was consistently associated with an increased risk of posterior circulation ischemia, including posterior circulation stroke (PCS), vertebrobasilar ischemia, and related cerebrovascular events. Large retrospective cohort studies by Hu et al. [36] demonstrated that VAH was independently associated with posterior rather than anterior circulation stroke, a finding further supported by Yang et al. [37] who reported an even stronger association in younger stroke populations. Similarly, Perren et al. [38] found a significantly higher frequency of VAH among patients with posterior circulation stroke, while Kulyk et al. [39] additionally identified an association with vertebrobasilar stenosis, suggesting that concomitant vascular pathology may further increase stroke risk.

These findings were reinforced by several case–control and multicenter studies. Gaigalaite et al. [40] reported a higher prevalence of VAH in patients with posterior circulation stroke and transient ischemic attack, whereas a decade later [41] demonstrated that the association is influenced by Circle of Willis anatomy, indicating a stronger effect in individuals with impaired collateral circulation. Li et al. [42] confirmed VAH as an independent predictor of posterior circulation infarction, while Chi et al. [50] demonstrated that VAH remained significantly associated with posterior circulation stroke after multivariable adjustment. Zhang et al. [44] further linked VAH with posterior circulation hypoperfusion and medullary infarction, particularly lateral medullary infarction accompanied by cerebellar involvement, supporting its hemodynamic importance.

Additional observational evidence from Vilimas et al. [43] showed that reduced diameter of the non-dominant vertebral artery was associated with an increased risk of posterior circulation stroke, particularly in patients younger than 65 years. Beyond ischemic stroke, Chuang et al. [45,47] reported significant associations between VAH and posterior circulation infarction, including PICA territory infarction, as well as vestibular neuropathy. Park et al. [46] similarly demonstrated a relationship between VAH and ipsilateral posterior circulation infarction, while Gilberti et al. [53] described frequent vertebrobasilar hypoplasia and VAH among patients with isolated midbrain infarction. The available evidence consistently supports VAH as an independent anatomical factor contributing to posterior circulation ischemia, with its clinical impact influenced by collateral circulation, vascular anatomy, and coexisting vertebrobasilar disease (Table 2).

### 3.3. Hemodynamic and Perfusion Mechanisms of Vertebral Artery Hypoplasia

The included studies (Supplementary Table S5) investigated the hemodynamic effects of VAH in healthy individuals, cerebrovascular patients, and subjects with vertebral artery disorders. VAH was assessed using ultrasonography, MRA, CTA, or hemodynamic criteria, while outcomes included vertebral artery flow, cerebrovascular reactivity, imaging performance, and associations with posterior circulation ischemia and vascular abnormalities [53,54,55,56,57,58,59].

Overall methodological quality (Supplementary Table S6) ranged from low to moderate risk of bias. Most studies used standardized imaging protocols and objective diagnostic criteria, although residual confounding, retrospective designs, and limited multivariable adjustment remained the main methodological limitations [53,54,55,56,57,58,59].

The included studies consistently demonstrated that VAH is associated with significant hemodynamic abnormalities affecting vertebral artery flow, cerebrovascular autoregulation, and posterior circulation perfusion. Experimental physiological studies by Sato et al. [53] showed that VAH is associated with reduced CO_2_ reactivity and impaired dynamic cerebrovascular regulation, suggesting diminished adaptive capacity of the posterior circulation despite partial compensation by the contralateral vertebral artery. Similarly, Chen et al. [55] demonstrated a strong relationship between vertebral artery diameter and blood flow, confirming that VAH significantly reduces both unilateral and total vertebral artery flow volume.

Clinical and diagnostic studies further supported these findings. Wang et al. [33] reported increased vascular resistance in patients with VAH and identified the resistance index (RI) as the most accurate Doppler parameter for detecting hemodynamic impairment. Likewise, Zarrintan et al. [56] demonstrated elevated RI values and asymmetric vertebral artery flow, while Chi et al. [50] found significantly lower vertebrobasilar flow volume and flow velocities in patients with VAH. Chi et al. [54] additionally showed that these abnormalities were independently associated with posterior circulation stroke and vertebrobasilar dolichoectasia, supporting a close interaction between vascular anatomy and cerebral perfusion. Mitsumura et al. [57] similarly confirmed an association between VAH, vertebral artery occlusion, and posterior circulation ischemia.

Mechanistic evidence was provided by Bao et al. [55] whose computational fluid dynamics analyses demonstrated reduced wall shear stress, increased oscillatory shear index, and disturbed flow patterns in hypoplastic vertebral arteries, suggesting endothelial dysfunction and vascular remodeling. Additional observational studies by Szárazová et al. [58] described altered collateral flow redistribution in posterior circulation stroke, whereas Hsu et al. [59] reported that bilateral VAH was associated with markedly reduced vertebrobasilar flow, earlier stroke onset, and more extensive infarction. Overall, the available evidence consistently indicates that VAH represents a hemodynamically significant vascular variant characterized by reduced flow capacity, impaired autoregulation, increased vascular resistance, and abnormal perfusion, thereby increasing susceptibility to posterior circulation ischemia (Table 3).

### 3.4. Vertebral Artery Hypoplasia and Specific Neurological Clinical Presentations: Non-Stroke and Mixed Syndromes

The included studies (Supplementary Table S7) investigated the clinical manifestations of VAH across patients with cerebrovascular disease, vertigo, vestibular syndromes, transient global amnesia, vertebrobasilar insufficiency, healthy controls, and individual case reports. VAH was assessed using Doppler ultrasonography, CTA, MRA, or DSA, while outcomes included posterior circulation ischemia, vestibular disorders, hemodynamic abnormalities, vascular anatomy, functional outcomes, and imaging-confirmed cerebrovascular events [60,61,62,63,64,65,66,67,68,69,70].

Overall methodological quality (Supplementary Table S8) ranged from low to high risk of bias depending on study design. Observational studies generally demonstrated standardized imaging protocols and appropriate statistical analyses, whereas residual confounding, heterogeneous diagnostic criteria, and the inherent limitations of case reports and small case series represented the main methodological concerns [60,61,62,63,64,65,66,67,68,69,70].

The included studies demonstrated that VAH is associated with a broad spectrum of non-stroke and mixed neurological manifestations involving transient, vestibular, brainstem, and vertebrobasilar insufficiency syndromes. Werner et al. [60] reported that VAH is associated with transient global amnesia through reduced posterior circulation perfusion and impaired cerebrovascular hemodynamics. Similarly, Zhang et al. [43] identified VAH as an independent factor associated with vertigo and posterior circulation ischemia, while Pasaoglu et al. [61] found a significantly higher prevalence of VAH among patients with central vertigo, supporting a vertebrobasilar origin of symptoms. Chuang et al. [45] further demonstrated a strong association between VAH and vestibular neuropathy with characteristic ipsilateral involvement, and Gaigalaite et al. [40] reported that VAH was associated not only with posterior circulation stroke but also with vertigo-related symptoms, suggesting overlap between ischemic and non-ischemic clinical presentations.

Evidence from posterior circulation disorders also supported the clinical relevance of VAH. Szárazová et al. [58] demonstrated that VAH may contributes to both embolic and hemodynamic mechanisms of posterior circulation ischemia, whereas Giannopoulos et al. [64] consistently observed ipsilateral VAH in patients with lateral medullary infarction. Luan et al. [63] described severe vertebrobasilar insufficiency resulting from the combination of VAH and contralateral vertebral artery stenosis, and Cai et al. [64] reported Bow Hunter syndrome associated with VAH, emphasizing susceptibility to dynamic compression-induced ischemia. In addition, Bae et al. [65] showed that VAH was associated with poorer functional outcomes and increased posterior circulation stroke risk in patients with atrial fibrillation-related ischemic stroke.

Additional case reports further expanded the clinical spectrum of VAH. Çiçek et al. [29] frequently identified VAH in patients presenting with headache, neck pain, and vertigo despite no association with arcuate foramen variants. Mestan [68] described bilateral VAH associated with cerebellar infarction after minor cervical trauma, Jianu et al. [69] reported pontine infarction in the presence of multiple congenital cerebrovascular anomalies including VAH, Vynichakis et al. [67] highlighted vertebrobasilar insufficiency following Jefferson fracture, and Ueki et al. [70] suggested that VAH may increase susceptibility to recurrent posterior circulation embolic stroke when combined with inherited thrombophilia. These findings indicate that VAH is associated with a wide range of neurological syndromes extending beyond ischemic stroke and may interact with anatomical, traumatic, and prothrombotic factors to increase posterior circulation vulnerability (Table 4).

### 3.5. Anatomical Characteristics, Vascular Variations, and Comorbid Cerebrovascular Associations of Vertebral Artery Hypoplasia

The included studies (Supplementary Table S9) investigated the anatomical and vascular characteristics associated with VAH in patients with posterior circulation ischemia, transient ischemic attack, cerebrovascular disease, healthy controls, and one anatomical case report. VAH was assessed using Doppler ultrasonography, CTA, MRA, or anatomical dissection, while outcomes included vascular anatomy, posterior circulation ischemia, vertebrobasilar abnormalities, stroke localization, and associations with other cerebrovascular variants [27,32,41,42,71,72].

Overall methodological quality (Supplementary Table S10) ranged from low to high risk of bias. Most observational studies used standardized imaging protocols and reliable exposure assessment, although incomplete adjustment for confounding factors and the inherent limitations of single-case reports represented the principal methodological concerns [27,32,41,42,71,72].

The included studies demonstrated that VAH is closely associated with a broad spectrum of cerebrovascular anatomical variants, vascular remodeling, and collateral circulation patterns that may influence posterior circulation vulnerability. Li et al. [42] showed that VAH is associated with progressive basilar artery (BA) bending, suggesting that chronic flow asymmetry contributes to downstream vascular remodeling and increases the risk of posterior circulation infarction. Similarly, Chi et al. [54] identified a strong relationship between VAH and vertebrobasilar dolichoectasia (VBD), supporting the concept that long-term hemodynamic alterations promote arterial elongation and tortuosity.

The interaction between VAH and collateral circulation was further emphasized by Gaigalaite et al. [41] who demonstrated that Circle of Willis (CoW) anatomy significantly modifies the clinical impact of VAH. In particular, the absence of bilateral posterior communicating arteries increased the risk of posterior circulation ischemia, whereas fetal-type CoW variants influenced the compensatory capacity of collateral blood flow. These findings highlight the importance of collateral reserve in determining the clinical consequences of VAH beyond arterial diameter alone.

Several studies also described important developmental and congenital vascular associations. Fortuniak et al. [27] reported that VAH is predominantly unilateral and frequently accompanied by V3 segment anomalies, reflecting variability in vertebral artery development. Rajasekhar et al. [71] described rare combinations of V4 hypoplasia and vertebral artery duplication, further illustrating the embryological diversity of posterior circulation anatomy. Layegh et al. [72] demonstrated that VAH commonly coexists with basilar artery hypoplasia and fetal posterior cerebral artery (PCA) variants, although these anatomical combinations were not consistently associated with infarct localization. Finally, Siva et al. [32] identified the V4 segment as particularly susceptible to hypoplastic changes and reported a significant association between V4 morphology and foramen transversarium asymmetry. These studies showed thats VAH represents part of a complex developmental and anatomical network involving collateral circulation, arterial remodeling, and vertebrobasilar morphology, all of which may contribute to posterior circulation vulnerability (Table 5).

## 4. Discussion

The available evidence suggests that vertebral artery hypoplasia should not be regarded solely as an incidental anatomical variant, but rather as a structurally and functionally relevant determinant of posterior circulation hemodynamics. Across heterogeneous imaging modalities and study populations, VAH consistently demonstrates a markedly reduced luminal diameter, typically below 2–2.2 mm, accompanied by decreased flow volume and pronounced vertebrobasilar asymmetry. Although reported prevalence varies widely depending on diagnostic criteria and imaging technique, the overall pattern across studies indicates that VAH is a frequent vascular variant with important physiological consequences rather than a rare anomaly [27,28,29,30,31,32,33,34,35].

From a clinical perspective, the most consistently reported association is between VAH and posterior circulation ischemia, including cerebellar and brainstem infarctions. Multiple cohort and case–control studies [36,37,38,39,40,50,51] have demonstrated that individuals with VAH have a significantly increased likelihood of posterior circulation stroke compared with those without VAH or with anterior circulation events. This association appears particularly strong in the presence of additional vascular risk factors such as hypertension, dyslipidemia, or concomitant vertebrobasilar pathology. Importantly, VAH has also been linked to specific ischemic patterns, including PICA territory infarction and lateral medullary syndromes [45,46,52] supporting a preferential vulnerability of posterior circulation subterritories to reduced vertebral inflow. Perfusion imaging studies further demonstrated delayed perfusion and relative hypoperfusion within the PICA territory even in patients without established infarction, indicating that VAH may produce chronic regional hemodynamic insufficiency before irreversible ischemic injury develops [73].

Beyond overt ischemic stroke, VAH is increasingly recognized in a broader spectrum of posterior circulation–related neurological syndromes. Patients with VAH have been described in association with central vertigo and vertebrobasilar insufficiency [43,61] transient ischemic attacks [40] and vestibular dysfunction [45] suggesting that hemodynamic compromise may manifest as both transient and persistent neurological symptoms. In several observational studies, VAH has also been more prevalent in populations presenting with nonspecific symptoms such as dizziness or headache [29] although the strength of causal inference in these settings remains limited. Collectively, these findings support the concept that VAH may contributes to a continuum of posterior circulation dysfunction rather than exclusively to frank infarction.

At the mechanistic level, VAH is strongly associated with chronic alterations in cerebral blood flow distribution and vascular resistance. Hemodynamic studies using Doppler ultrasound, CTA, and advanced flow imaging consistently demonstrate reduced unilateral vertebral flow, increased resistance indices, and compensatory redistribution of flow toward the contralateral vertebral artery [31,33,50,56]. While global cerebral perfusion may be preserved under baseline conditions, compensatory mechanisms appear increasingly limited with physiological stress and aging. Supporting this concept, 4D flow MRI demonstrated that VAH modifies the normal age-related redistribution of cerebral blood flow, with preservation of basilar artery flow occurring at the expense of reduced anterior circulation perfusion, suggesting chronic compensatory adaptation of cerebral hemodynamics [74]. Computational fluid dynamics further suggest that VAH may induce disturbed flow patterns characterized by reduced wall shear stress, increased oscillatory shear index, and prolonged residence time, all of which are known contributors to endothelial dysfunction and vascular remodeling [55].

These hemodynamic disturbances provide a plausible biological framework linking VAH not only to ischemic susceptibility but also to arterial pathology such as dissection and progressive vascular remodeling. Reduced flow velocity within a hypoplastic artery may promote endothelial injury, impaired nitric oxide signaling, and a prothrombotic vascular environment, thereby potentially increasing susceptibility to both thromboembolic events and structural vessel wall damage. Consistent with this concept, VAH has been reported in association with vertebral artery dissection in both adult and pediatric populations [75] particularly in situations involving mechanical stress such as cervical rotation or sports-related neck movements, suggesting that anatomical vulnerability may amplify the impact of external triggers. Earlier clinical observations also suggested that abnormal hemorheological properties may further amplify the clinical consequences of VAH, with symptomatic patients demonstrating greater blood viscosity abnormalities than asymptomatic individuals, indicating that impaired blood rheology may act synergistically with congenital low-flow anatomy [76]. Although VA hypoplasia has predominantly been investigated as a risk factor for posterior circulation ischemic events, its relationship with hemorrhagic complications remains insufficiently explored. Some reports of fusiform vertebral artery aneurysms suggest that altered flow dynamics and reduced contralateral vertebral artery capacity may influence aneurysm behavior; nevertheless, current evidence is insufficient to establish whether VA hypoplasia modifies hemorrhagic risk [77].

An additional key dimension of VAH pathophysiology lies in its interaction with the broader cerebrovascular anatomical network. The clinical expression of VAH is strongly modulated by collateral circulation, particularly the configuration of the Circle of Willis and the presence of posterior communicating arteries [40,78]. Individuals with incomplete collateral pathways exhibit reduced capacity to compensate for unilateral vertebral hypoplasia, thereby increasing dependence on the dominant vertebral artery and lowering the threshold for ischemia. Moreover, VAH frequently coexists with other developmental vascular variants, including basilar artery hypoplasia and fetal-type posterior cerebral circulation [32,72], collectively reflecting a spectrum of congenital vascular remodeling that shapes posterior circulation resilience. Supporting this developmental concept, anomalous vertebral artery origin and abnormal transverse foraminal entry have been independently associated with smaller vertebral artery diameter, suggesting that several congenital vertebral artery variants likely represent different manifestations of impaired embryological development rather than isolated anatomical abnormalities [78,79]. Similarly, the reported coexistence of VAH with a high jugular bulb further supports the concept that congenital vascular variants of the posterior cranial circulation may share common developmental mechanisms, although the clinical significance of this association remains uncertain [80].

Taken together, the accumulated evidence supports a unified interpretation of VAH as a congenital anatomical phenotype that may confer a low-flow hemodynamic state. Rather than acting as an isolated anatomical finding, VAH operates within a complex interplay of hemodynamic stress, collateral capacity, vascular wall biology, and acquired risk factors. Many individuals with VAH appear to remain clinically asymptomatic, presumably because of compensatory mechanisms; however, when these mechanisms are overwhelmed by aging, atherosclerosis, anatomical constraints, or acute triggers such as dissection, the risk of posterior circulation ischemia and related neurological syndromes may increases substantially. This integrative perspective positions VAH as a clinically meaningful context-dependent low-flow vascular susceptibility phenotype with relevance across the spectrum of cerebrovascular disease. Accordingly, VAH should be viewed as a vascular susceptibility factor rather than a deterministic cause of cerebrovascular disease, with clinical expression depending on the interaction between congenital anatomy, collateral capacity, aging, and acquired vascular risk factors.

Importantly, direct comparison with the previous reviews by Katsanos and colleagues highlights both substantial agreement and important differences in scope. The 2013 comprehensive review synthesized case reports, imaging studies, and cohort data and proposed that VAH may predispose to posterior circulation ischemia through reduced vertebral flow, impaired compensation, and interaction with conventional vascular risk factors, but it did not provide a quantitative pooled estimate or formal methodological quality assessment [81]. The subsequent 2017 systematic review and meta-analysis included eight observational cohort studies comprising 3875 patients with acute ischemic stroke and demonstrated that VAH was significantly more prevalent among patients with posterior than anterior circulation ischemia (pooled risk ratio [RR] 2.12, 95% CI 1.60–2.82). After exclusion of an outlying study, the association remained significant (RR 1.81, 95% CI 1.58–2.06), while heterogeneity decreased from I^2^ = 75% to I^2^ = 0%, indicating that differences in study populations and diagnostic approaches substantially influenced the pooled estimate [82]. The principal sources of heterogeneity identified in that meta-analysis were variation in VAH definitions (diameter thresholds ranging from 2 to 3 mm, with asymmetry criteria used in some studies), imaging modalities, demographic characteristics, and study populations. The present systematic review is concordant with the overall conclusion that VAH is associated with posterior circulation ischemic vulnerability, but it extends prior work in several important respects. By synthesizing 46 studies, it incorporates newer cohort and imaging studies together with quantitative hemodynamic investigations, collateral circulation, vascular remodeling, computational fluid dynamics, and non-stroke neurological manifestations that were not comprehensively integrated in the previous meta-analysis. Importantly, this expanded evidence base includes the recent longitudinal cohort by Li et al., which followed 1464 patients for approximately 20 years and found that VAH was independently associated with new-onset posterior circulation infarction (OR 1.481, 95% CI 1.019–2.003), with progression of basilar artery bending identified as a potential intermediate mechanism. Notably, approximately 50.2% of the association was estimated to be mediated through basilar artery bending, although the wide confidence interval indicates substantial uncertainty. This study provides longitudinal support for the hemodynamic and vascular-remodeling mechanisms identified in earlier cross-sectional and computational studies and further strengthens the interpretation of VAH as a dynamic marker of posterior circulation vulnerability rather than an isolated anatomical variant [42]. No major qualitative divergence was identified regarding the association between VAH and posterior circulation ischemia; however, the present review extends this conclusion to additional phenotypes and mechanistic domains that were not addressed in the earlier pooled analyses. These additional evidence domains support a more refined interpretation of VAH as a context-dependent low-flow vascular susceptibility phenotype whose clinical expression depends on the interaction among reduced vertebral inflow, collateral capacity, coexisting vascular abnormalities, and acquired cerebrovascular risk factors. Regarding hemorrhagic posterior circulation events, this review found no clear evidence of an association between VAH and cerebellar or brainstem hemorrhage. This may partly reflect the predominantly ischemic focus and observational design of the available literature. Although isolated reports and hemodynamic considerations warrant further investigation, the relationship between VAH and posterior circulation hemorrhage remains insufficiently characterized and should be specifically addressed in future studies. Thus, rather than simply reaffirming an association with posterior circulation stroke, the present review provides an evidence-informed mechanistic framework that helps explain why VAH remains clinically silent in many individuals while contributing to posterior circulation vulnerability in others.

From a clinical perspective, the findings of the present review suggest that incidentally detected vertebral artery hypoplasia should be interpreted within the broader context of an individual’s cerebrovascular anatomy and vascular risk profile rather than as an isolated imaging finding. Current evidence does not support routine screening, additional diagnostic investigations, or preventive treatment solely on the basis of incidentally identified vertebral artery hypoplasia in otherwise asymptomatic individuals. Instead, awareness of this vascular variant may contribute to a more individualized interpretation of posterior circulation symptoms, particularly in patients presenting with additional vascular abnormalities, impaired collateral circulation, or established cerebrovascular risk factors. Future prospective studies should establish standardized diagnostic criteria integrating vessel diameter with quantitative flow-based measurements, evaluate collateral circulation using advanced multimodal imaging, and determine whether vertebral artery hypoplasia provides incremental prognostic value in cerebrovascular risk stratification beyond conventional vascular risk factors.

**Figure 2:**
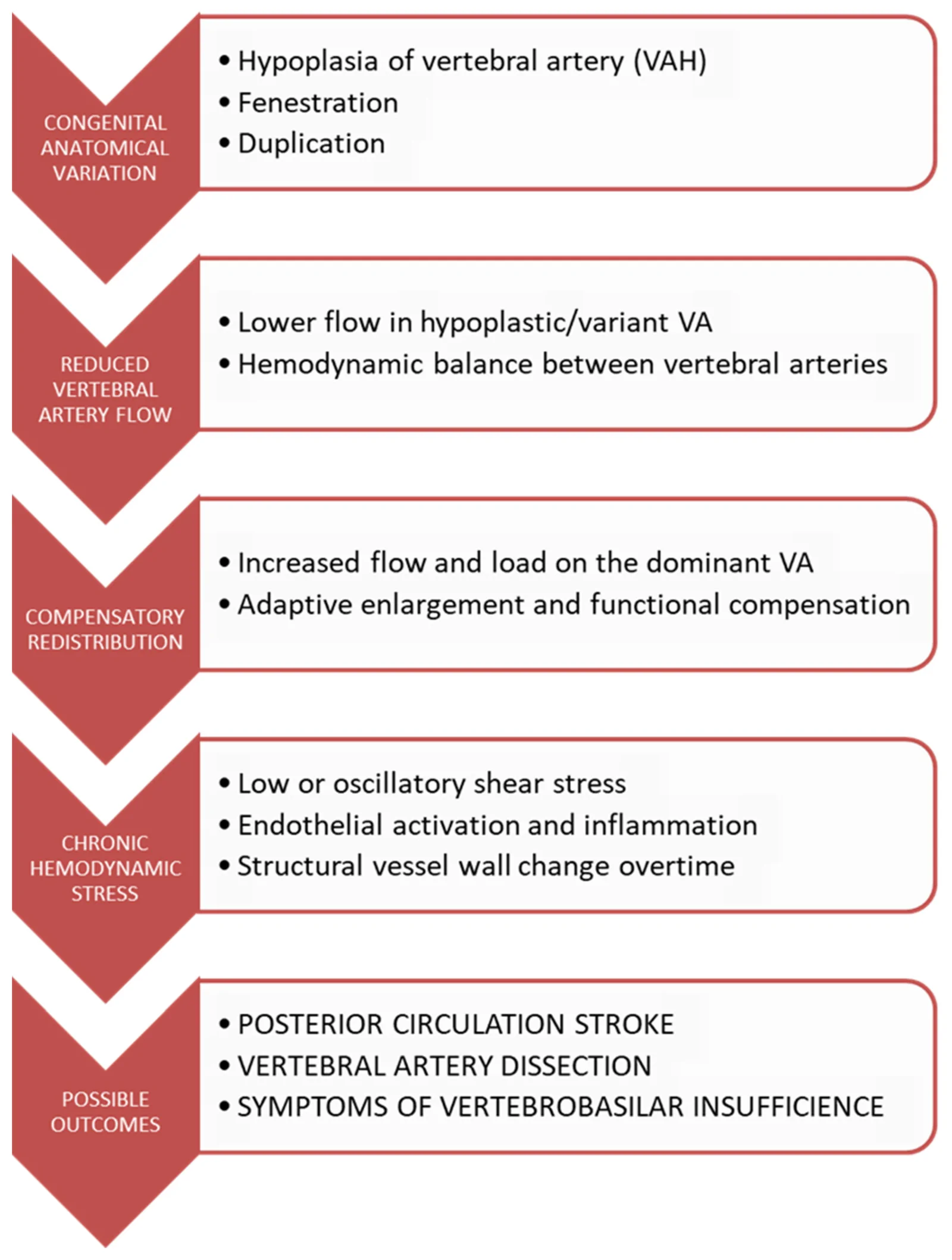
Conceptual model based on the systematic synthesis of currently available evidence.

Integrating current findings, we propose a conceptual model that depicts the potential sequence of events linking VAH with altered cerebral hemodynamics, compensatory vascular responses, and subsequent clinical consequences (Figure 2).

The proposed model should not be interpreted as direct causal evidence but rather as a conceptual framework integrating the findings identified across heterogeneous observational, anatomical, imaging, and hemodynamic studies included in the present systematic review.

Nevertheless, several limitations should be acknowledged. The included literature is highly heterogeneous with respect to VAH definitions, ranging from fixed diameter thresholds to flow-based and asymmetry criteria, which limits direct comparability across studies and introduces significant variability in reported prevalence and effect size. A substantial proportion of available evidence originates from retrospective or single-center designs, with residual confounding and selection bias remaining incompletely addressed. Even in larger cohort studies, VAH is often analyzed as a binary variable, which may oversimplify a continuous spectrum of vertebrobasilar hypoplasia and underrepresent dose–response relationships between vessel caliber and clinical outcomes. Although mechanistic studies strongly support hemodynamic plausibility, causal inference remains limited, particularly in non-stroke phenotypes such as vertigo or headache, where symptom attribution is less specific. The search was expanded to include the Cochrane Library and Wiley Online Library; however, Embase and Web of Science were not searched, which may have resulted in the omission of some relevant studies.

In contrast to these limitations, the strength of the present synthesis lies in its integrated multi-layered approach, combining epidemiological associations, anatomical variability, hemodynamic evidence, and computational modeling into a unified framework. This allows a more comprehensive interpretation of VAH as a dynamic vascular phenotype rather than a static anatomical finding. The consistency of findings across independent methodological domains, clinical stroke cohorts, Doppler flow studies, imaging-based anatomical analyses, and CFD simulations, further strengthens the internal validity of the proposed pathophysiological model.

## 5. Conclusions

In conclusion, the accumulated evidence suggests that VAH should be conceptualized not simply as an incidental anatomical finding, but as a potential marker of vertebrobasilar hemodynamic vulnerability. Future research should prioritize international consensus regarding standardized diagnostic thresholds that combine vessel diameter and quantitative flow parameters rather than relying exclusively on fixed diameter criteria. Prospective longitudinal studies integrating CTA/MRA, Doppler ultrasonography, 4D-flow MRI, collateral circulation assessment, and computational hemodynamic analyses are needed to determine whether VAH independently improves cerebrovascular risk prediction beyond established vascular risk factors. Such studies should also evaluate whether incorporation of VAH into future posterior circulation risk-stratification models provides incremental prognostic value.

## Figures and Tables

**Table 1:** Summary of studies investigating the prevalence and epidemiology of vertebral artery hypoplasia.

Study	Country	Year	Study Design	Sample Size	Population	VAH Definition	VAH Prevalence	Key Findings	Level of Evidence
Fortuniak et al. [27]	Poland	2016	Retrospective cross-sectional CTA	1800	Adults undergoing CTA	VA ≤ 50% contralateral VA	20.0%	VAH unilateral; common finding in CTA population; discussed as risk marker for posterior circulation ischemia	IV
Peterson et al. [28]	Canada	2010	Retrospective MRI cross-sectional	131	Patients with neck pain	Visual MRI grading	43.5%	High prevalence of VAH; right-sided predominance; more frequent in women; good MRI reliability	IV
Çiçek et al. [29]	Turkey	2022	Retrospective cross-sectional	139	Patients with headache, neck pain and dizziness	V4 diameter criteria (MRA/CTA)	32.4%	Moderate prevalence; no significant association between VAH and arcuate foramen	IV
Min & Lee [30]	Republic of Korea	2007	Cross-sectional diagnostic study	410	Healthy adults	VA < 50% contralateral	23.9%	VAH common anatomical variant; contralateral compensatory flow increase	IV
Chen et al. [31]	Taiwan	2010	Ultrasound cohort study	1000	General adult population	VA diameter ~<2.5–2.6 mm + flow criteria	3.3–5.9%	Strong correlation between VA diameter and flow volume; reduced net vertebral flow in VAH	III
Siva et al. [32]	India	2026	Retrospective cross-sectional imaging study	151	Cerebrovascular CTA patients	VA ≤ 2 mm	47.0%	High VAH prevalence; V4 segment most vulnerable; strong anatomic variability	IV
Wang et al. [33]	China	2020	Retrospective cross-sectional diagnostic accuracy study	138	Dizziness/headache patients	VA ≤ 2.5 mm or asymmetry ratio	Not explicitly stated	RI most accurate parameter for detecting VA pathology; VAH subgroup identified	IV
Vilimas et al. [34]	Lithuania	2022	Case–control observational	742	Stroke patients + controls	Non-dominant VA < 3 mm	~56% (<3 mm controls), higher in PCI	Smaller VA independently associated with posterior circulation stroke risk	III
Li et al. [35]	China	2015	Retrospective imaging study	110	TIA, stroke, controls	VA < 2–3 mm	40.6% (vertebrobasilar TIA group)	VAH significantly more frequent in vertebrobasilar TIA; associated with multivessel disease	III

**Table 2:** Summary of studies investigating the association between vertebral artery hypoplasia and the risk of posterior circulation stroke.

Study	Country	Year	Study Design	Sample Size	Population	VAH Definition	VAH Prevalence	Outcome	Key Findings	Level of Evidence
Hu et al. [36]	China	2013	Retrospective cohort	841	AIS (ACI + PCI)	VA < 2 mm (CTA/MRA)	10.8%	PCI vs. ACI	VAH independent anatomical factor for posterior circulation stroke (OR ≈ 2.0)	III
Yang et al. [37]	China	2016	Case–control	235	AIS < 50 years	VAH < 2 mm (DSA)	16.1%	PCI vs. ACI	VAH independently associated with PCI (OR ≈ 2.21)	III
Perren et al. [38]	Switzerland	2007	Retrospective cohort	725	First-ever AIS	VA ≤ 2.5 mm/asymmetry	13% (PCI group)	Stroke territory	VAH significantly more frequent in PCI (13% vs. 4.6%)	III
Kulyk et al. [39]	Italy	2018	Retrospective observational	750	AIS patients	US-defined VAH	33.7% (PCI vs. 14.1% ACI)	PCI	VAH significantly associated with posterior circulation stroke; OR up to 3.2 for vascular stenosis	III
Gaigalaite et al. [40]	Lithuania	2016	Case–control	1109	PCS/TIA + controls	VA < 3 mm	58.3% (PCS/TIA)	PCS/TIA	VAH more frequent in PCS/TIA; OR ~1.8 for posterior ischemic events	III
Gaigalaite et al. [41]	Lithuania	2026	Case–control	1339	PCS/TIA + controls	CTA/MRA VAH criteria	27.0%	PCS/TIA	Significant VAH × CoW interaction; increased risk when posterior collateral pathways absent	III
Li et al. [42]	China	2026	Retrospective multicenter longitudinal cohort	1464	Neurological patients	VA < 2 mm or asymmetry	37.4%	POCI	VAH independently associated with posterior circulation infarction (OR 1.48)	III
Vilimas et al. [34]	Lithuania	2022	Case–control	742	Stroke + controls	Non-dominant VA < 3 mm	NA	PCI/VAS	Smaller VA significantly associated with PCI; strongest in <65 years (OR up to 4.12)	III
Zhang et al. [43]	China	2017	Retrospective observational	224	Vertigo + vascular risk	VA ≤ 2 mm/asymmetry	26%	PCI occurrence	VAH independently associated with PCI (OR ~2.6–2.7)	III
Zhang et al. [44]	China	2016	Imaging cohort	172	Suspected stroke	VA ≤ 2 mm/asymmetry	33.1%	PCI + perfusion deficit	VAH associated with PCI (OR 2.10) and posterior hypoperfusion	III
Chuang et al. [45]	Taiwan	2011	Case–control	119/69	Vestibular neuropathy	VA < 2.2 mm + asymmetry	42% (VN group)	Neurologic dysfunction	VAH associated with vestibular neuropathy (RR 2.2); ipsilateral effect common	III
Park et al. [46]	Republic of Korea	2007	Case–control	835	Stroke + controls	VA < 2 mm	35.2% stroke/26.5% controls	Stroke territory	VAH significantly associated with posterior circulation stroke and ipsilateral infarcts	III
Chuang et al. [47]	Taiwan	2006	Matched case–control	382/191 stroke	AIS + controls	VA < 2 mm	11.5% stroke vs. 2.1% controls	Stroke location	VAH strongly associated with posterior circulation stroke (esp. PICA territory)	III
Dinç et al. [48]	Turkey	2021	Retrospective cohort	609	AIS	VA diameter asymmetry > 2 mm	37%	PCI + stenosis	VAH independently associated with posterior atherosclerotic stenosis and PCI	II
Bakalarz et al. [49]	Poland	2022	Retrospective observational	240	Neurology inpatients	VA < 2 mm	3.2%	Stroke/TIA/vertigo	VAH associated with cerebellar ischemia (10%) and vertigo (27.5%)	III
Chi et al. [50]	Taiwan	2019	Retrospective observational	353	AIS (posterior vs. anterior)	VA ≤ 2.2 mm + low-flow criteria	44.8% PCI vs. 22.4% ACI	Posterior stroke	VAH independently associated with posterior circulation stroke; reduced vertebrobasilar flow and abnormal Doppler parameters supported its hemodynamic relevance	III
Zhang et al. [51]	China	2021	Multicenter retrospective cohort	179	Medullary infarction	VA < 2.5 mm + asymmetry; V4 hypoplasia	42.5%	Medullary infarction subtype	VAH/V4 hypoplasia particularly frequent in lateral medullary infarction and lateral medullary infarction with cerebellar involvement	III
Gilberti et al. [52]	Italy	2014	Case series	9	Isolated midbrain infarction	VA < 2 mm or >50% asymmetry	44.4%	Midbrain infarction	VAH and vertebrobasilar hypoplasia were frequently observed among isolated midbrain infarction cases, suggesting a possible anatomical predisposition to mesencephalic ischemia	IV

**Table 3:** Summary of studies investigating the hemodynamic and perfusion mechanisms associated with vertebral artery hypoplasia.

Study	Country	Year	Study Design	Sample Size	Population	VAH Definition	Hemodynamic Parameters	Key Findings	Level of Evidence
Sato et al. [53]	Japan	2015	Controlled human physiological study	28	Healthy adults	VA flow ≤ 40 mL/min	CO_2_ reactivity, VA flow	VAH reduces CO_2_ reactivity; impaired dynamic cerebrovascular regulation, partially compensated by contralateral VA	III
Chen et al. [31]	Taiwan	2010	Ultrasound cohort	1000	General population	Diameter + flow criteria	VA flow volume, net flow	Strong diameter–flow correlation; VAH significantly reduces unilateral and net vertebral flow	III
Wang et al. [33]	China	2020	Retrospective cross-sectional diagnostic accuracy study	138	Patients with dizziness/headache	VA ≤ 2.5 mm/asymmetry ratio	PSV, EDV, RI	RI showed highest diagnostic accuracy; VAH associated with altered resistance and hemodynamic disturbance	IV
Chi et al. [54]	Taiwan	2019	Retrospective cohort	465	AIS patients	VA ≤ 2.2 mm/flow ≤ 30 mL/min	Flow volume, VBD patterns	VAH strongly associated with vertebrobasilar dolichoectasia and reduced flow; marked asymmetry of posterior circulation perfusion	III
Bao et al. [55]	China	2023	Computational mechanistic study based on human CTA data	14	sVAD + VAH	Imaging-defined VAH	WSS, OSI, RRT, velocity	VAH associated with low wall shear stress, high oscillatory shear index and disturbed flow patterns in dissection segments	NA
Zarrintan et al. [56]	Iran	2021	Doppler case–control	30	VAH vs. controls	VA ≤ 2.1 mm	PSV, RI	VAH associated with increased RI and asymmetric flow patterns, indicating increased vascular resistance	III
Mitsumura et al. [57]	Japan	2016	Retrospective cohort	129	AIS patients	VA < 2.5–3.0 mm + flow criteria	VA occlusion, flow status	VAH independently associated with VA occlusion and posterior circulation ischemia; stronger effect in posterior stroke group	III
Szárazová et al. [58]	Slovakia	2015	Prospective observational	80	Posterior circulation stroke	VA ≤ 2.5 mm + Doppler criteria	PSV, RI, flow redistribution	VAH associated with altered flow redistribution and embolic/hemodynamic stroke mechanisms	II
Hsu et al. [59]	Taiwan	2021	Retrospective cross-sectional	467	AIS patients	VA ≤ 2.2 mm/flow ≤ 30 mL/min	Flow volume, stroke distribution	Bilateral VAH associated with markedly reduced flow, younger stroke onset and multi-territory infarction	III
Chi et al. [50]	Taiwan	2018	Retrospective observational	353	AIS (posterior vs. anterior stroke)	VA ≤ 2.2 mm + low-flow criteria	Flow volume, velocity, ROC analysis	VAH associated with significantly reduced vertebrobasilar flow volume and lower flow velocity; Doppler parameters accurately identified hemodynamically significant VAH associated with posterior circulation stroke	III

**Table 4:** Summary of studies investigating specific neurological clinical presentations associated with vertebral artery hypoplasia.

Study	Country	Year	Study Design	Sample Size	Population	VAH Definition	Clinical Presentation	Key Findings	Level of Evidence
Werner et al. [60]	Germany	2024	Case–control	206	TGA patients	VA diameter < 2–2.5 mm	Transient global amnesia	VAH associated with reduced posterior circulation perfusion and altered hemodynamics; possible role in TGA pathophysiology	III
Zhang et al., (vertigo cohort) [43]	China	2017	Retrospective observational	224	Isolated vertigo patients	VA ≤ 2 mm/asymmetry	Vertigo	VAH independently associated with posterior circulation ischemia (OR ~2.6–2.7)	III
Pašaoglu et al. [61]	Turkey	2017	Case–control	249	Central vertigo patients	VA < 2 mm/stenosis criteria	Central vertigo	Higher VAH prevalence in symptomatic group; strong association with vertebrobasilar pathology	III
Chuang et al. [45]	Taiwan	2011	Case–control	119	Vestibular neuropathy	VA < 2.2 mm + asymmetry	Vestibular neuropathy	VAH significantly more frequent in VN; ipsilateral dominance pattern observed	III
Gaigalaite et al. [40]	Lithuania	2026	Case–control	1109	PCS/TIA + controls	VA < 3 mm	Vertigo + PCS/TIA	VAH associated with posterior ischemic events and vertigo-like symptoms in cerebrovascular population	III
Szárazová et al. [58]	Slovakia	2015	Prospective observational	80	PCS patients	VA ≤ 2.5 mm + Doppler criteria	Posterior circulation stroke syndromes	VAH associated with embolic/hemodynamic mechanisms contributing to PCS clinical spectrum	II
Giannopoulos et al. [62]	Greece	2007	Case series	3	Lateral medullary syndrome	Imaging-confirmed VAH	Medullary syndrome	VAH ipsilateral to ischemic territory; suggests direct hypoperfusion mechanism	IV
Luan et al. [63]	Multinational	2025	Case report	1	Vertebrobasilar insufficiency	Contralateral VA hypoplasia	Syncope, vertigo, VBI	Severe V4 stenosis + VAH caused critical vertebrobasilar insufficiency; resolved after intervention	V
Cai et al. [64]	USA	2018	Case report	1	Bow Hunter syndrome	Pre-existing VAH	Dynamic vertebrobasilar insufficiency	VAH + dynamic compression caused positional VBI; surgical decompression effective	V
Bae et al. [65]	Republic of Korea	2023	Retrospective cohort	407	AF-related AIS	VA ≤ 2 mm	Stroke outcome + posterior circulation stroke	Left VAH associated with posterior stroke, basilar artery occlusion and worse functional outcome	III
Chung & Sovory [66]	USA	2022	Case report	1	Lateral medullary syndrome	CTA-confirmed VAH	Wallenberg syndrome	VAH present without atherosclerosis; suggests primary hemodynamic ischemic mechanism	V
Çiçek et al. [29]	Turkey	2022	Retrospective cross-sectional	139	Patients with headache, neck pain and vertigo	MRA/CTA (V4)	Headache, vertigo	VAH common in symptomatic patients but not significantly associated with arcuate foramen; supports anatomical contribution to vertebrobasilar symptoms	IV
Vynichakis et al. [67]	Greece	2019	Case report	1	Jefferson fracture	CTA/MRA-confirmed VAH	Post-traumatic vertigo	VAH increased vertebrobasilar vulnerability after cervical trauma	V
Mestan [68]	USA	1999	Case report	1	Posterior fossa ischemia	Bilateral angiographic VAH	Cerebellar infarction	Minor cervical trauma plus bilateral VAH associated with posterior fossa ischemia	V
Jianu et al. [69]	Romania	2018	Case report	1	Pontine infarction	CTA-confirmed VAH	Brainstem ischemia	Multiple congenital vascular anomalies including VAH associated with vertebrobasilar stroke	V
Ueki et al. [70]	Japan	2020	Case report	1	Posterior embolic stroke	MRA-defined VAH	Recurrent embolic stroke	VAH combined with inherited protein C deficiency contributed to recurrent posterior circulation embolism	V

**Table 5:** Summary of studies investigating anatomical features, associated vascular variants, and cerebrovascular comorbidities in patients with vertebral artery hypoplasia.

Study	Country	Year	Study Design	Sample Size	Population	VAH Definition	Anatomical/Vascular Variable	Key Findings	Level of Evidence
Li et al. [42]	China	2026	Retrospective multicenter longitudinal cohort	1464	Neurological patients	VA < 2 mm/asymmetry	Basilar artery bending (BA bending)	VAH associated with progressive BA bending; mediates risk of posterior circulation infarction (~50%)	III
Gaigalaite et al. [41]	Lithuania	2026	Case–control	1339	PCS/TIA + controls	MRA/CTA VAH	Circle of Willis (CoW), fetal variants	Significant VAH × CoW interaction; absence of bilateral PComAs increases stroke risk; fetal CoW effect modified by VAH	III
Fortuniak et al. [27]	Poland	2016	CTA cross-sectional	1800	CTA population	VA ≤ 50% contralateral	V3 segment anomalies	VAH always unilateral; associated with rare V3 anomalies and collateral circulation variations	IV
Rajasekhar et al. [71]	India	2017	Cadaveric case report	1	Anatomical specimen	Anatomical VAH	Posterior circulation anatomy	Rare combined V4 hypoplasia and arterial duplication; highlights embryologic variability	V
Layegh et al. [72]	Iran	2024	Retrospective cross-sectional	155	PCS patients	VA ≤ 2 mm	Basilar artery hypoplasia + fetal PCA	Strong association between VAH and BA hypoplasia; fetal PCA frequently coexists; no direct infarct localization effect	IV
Chi et al. [54]	Taiwan	2019	Retrospective cohort	465	Stroke patients	VA ≤ 2.2 mm/flow criteria	Vertebrobasilar dolichoectasia (VBD)	VAH strongly associated with VBD and flow asymmetry; synergistic effect on posterior stroke risk	III
Siva et al. [32]	India	2026	Retrospective CTA	151	CTA vascular evaluation	VA ≤ 2 mm	Segmental anatomy (V4 segment)	V4 segment most vulnerable; significant correlation between V4 morphology and foramen transversarium asymmetry	IV

## Data Availability

All data is contained within article.

## Supplementary Materials

The following supporting information can be downloaded at: https://www.mdpi.com/article/10.3390/neurolint18090169/s1, among the mentioned supplementary tables, the PRISMA checklist is available as well as the Supplementary Materials (Supplementary Table S11). Supplementary Table S1. Characteristics of studies investigating the overall clinical and vascular characteristics of vertebral artery hypoplasia; Supplementary Table S2. Quality assessment of studies included in the general VAH evidence domain; Supplementary Table S3. Characteristics of studies evaluating the association between vertebral artery hypoplasia and posterior circulation ischemia; Supplementary Table S4. Quality assessment of studies investigating VAH and posterior circulation ischemia; Supplementary Table S5. Characteristics of studies assessing hemodynamic alterations associated with vertebral artery hypoplasia; Supplementary Table S6. Quality assessment of studies evaluating the hemodynamic consequences of VAH; Supplementary Table S7. Characteristics of studies investigating clinical manifestations associated with vertebral artery hypoplasia; Supplementary Table S8. Quality assessment of studies evaluating clinical manifestations of VAH; Supplementary Table S9. Characteristics of studies assessing anatomical and vascular variations associated with vertebral artery hypoplasia; Supplementary Table S10. Quality assessment of studies evaluating anatomical and vascular characteristics associated with VAH; Supplementary Table S11. PRISMA 2020 Checklist.
