# Supplementary material for: Vertebral Artery Hypoplasia and Posterior Circulation Vulnerability: A Systematic Review of Epidemiological, Hemodynamic, and Clinical Evidence
Source: Neurol Int. 2026 Sep 1;18(9):169. doi: 10.3390/neurolint18090169 (PMC13610102; doi:10.3390/neurolint18090169)
Supplement: Supplementary file 1 [file neurolint-18-00169-s001.zip › neurolint-4477234-supplementary.pdf]

Supplementary Table S1. Characteristics of studies investigating the overall clinical and vascular characteristics of vertebral artery hypoplasia

Study	Population (P)	Exposure (E)	Comparison (C)	Outcomes (O)
Fortuniak et al. [27]	AIS patients (n=725)	VAH (CTA criteria)	No VAH	Posterior stroke, RI
Peterson et al., [28]	Neck pain MRI pts	VAH (visual MRI)	Non-VAH	Prevalence, lateralization
Çiçek et al [29]	Patients with headache, neck pain, and dizziness	VAH (MRA/CTA V4 criteria) with arcuate foramen assessment	Patients without VAH and without arcuate foramen variation	VAH prevalence, anatomical variations, VAH–arcuate foramen association
Min & Lee [30]	Healthy adults	VAH (MRA)	Normal VA	Flow asymmetry
Chen et al. [31]	General population	VAH (US flow)	Non-VAH	Flow volume
Siva et al. [32]	CTA patients	VAH ≤2 mm	Normal VA	Anatomical variability
Wang et al. [33]	Symptomatic pts	VAH (US criteria)	Stenosis vs normal	RI, PSV
Vilimas et al. [34]	Stroke + controls	VAH <3 mm	Controls	PCI risk
Li et al. [35]	TIA, ischemic stroke patients, healthy controls	VAH, MRA diameter <2–3 mm	Healthy controls; TIA vs ischemic stroke; vertebrobasilar TIA vs ICA TIA	VAH prevalence, association with vertebrobasilar TIA, severity of cranial/cervical arterial stenosis, DWI ischemic lesions, vascular morphology

Supplementary Table S2. Quality assessment of studies included in the general VAH evidence domain

Study	Q1 Inclusion criteria	Q2 Study subjects	Q3 Exposure measured	Q4 Standard criteria	Q5 Confounders identified	Q6 Confounding addressed	Q7 Outcomes measured	Q8 Statistical analysis	Overall RoB
Fortuniak et al. [27]	Yes	Yes	Yes	Yes	Yes	Partial	Yes	Yes	Low
Peterson et al., [28]	Yes	Yes	Partial	No	No	No	Yes	Yes	Moderate
Çiçek et al [29]	Yes	Yes	Yes	Yes	No	No	Yes	Yes	Moderate
Min & Lee [30]	Yes	Yes	Yes	Yes	Partial	No	Yes	Yes	Moderate
Chen et al. [31]	Yes	Yes	Yes	Yes	Partial	No	Yes	Yes	Moderate
Siva et al. [32]	Yes	Yes	Yes	Yes	Partial	Partial	Yes	Yes	Moderate
Wang et al.	Yes	Yes	Yes	Yes	Partial	Partial	Yes	Yes	Moderate

[33]									
Vilimas et al. [34]	Yes	Yes	Yes	Yes	Yes	Yes	Yes	Yes	Low
Li et al. [35]	Yes	Yes	Yes	Yes	Partial	No	Yes	Yes	Moderate

Supplementary Table S3. Characteristics of studies evaluating the association between vertebral artery hypoplasia and posterior circulation ischemia

Study	Population (P)	Exposure (E)	Comparison (C)	Outcomes (O)
Hu et al. [36]	AIS patients (n=841)	VAH <2 mm	No VAH	PCI, TOAST subtype, posterior stenosis
Yang et al [37]	Young AIS patients	VAH <2 mm (DSA)	No VAH	PCI vs ACI, OR for PCI
Perren et al. [38]	First AIS patients (n=725)	VAH (US criteria)	No VAH	Posterior stroke, RI, recurrence
Kulyk et al. [39]	First AIS patients (n=750)	VAH	No VAH	PCI, basilar stenosis, fetal PCA
Gaigalaite et al. [40]	PCS/TIA patients + controls	VAH <3 mm	Controls	PCS/TIA risk, VA stenosis
Gaigalaite et al. [41]	PCS/TIA patients + controls	VAH + CoW variants	Controls	PCS/TIA, CoW interaction
Li et al. [42]	Neurological patients	VAH	No VAH	BA bending, recurrent POCI
Vilimas et al. [34]	Stroke patients + controls	VAH <3 mm	ACI, controls	PCI risk, prevalence
Zhang et al. [43]	Vertigo patients	VAH	No VAH	PCI, posterior stenosis
Zhang et al. [44]	Suspected stroke patients	VAH	No VAH	Perfusion deficits, PCI
Chuang et al [45]	Vestibular neuritis + controls	VAH	Controls	VAH prevalence, vestibular ischemia
Park et al. [46]	AIS patients + controls	VAH <2 mm	Controls	PCS frequency, lesion distribution
Chuang et al., [47]	Stroke patients + matched controls	VAH <2 mm	Controls	Posterior stroke, flow volume
Dinç et al., [48]	AIS patients	VAH	No VAH	PCI, posterior stenosis
Bakalarz et al., [49]	VAH, SVAA and controls	VAH <2 mm	Controls	Stroke, TIA, vertigo
Chi et al. [50]	AIS patients (posterior vs anterior stroke)	VAH ≤2.2 mm + low flow	Posterior vs anterior stroke; VAH vs non-VAH	Posterior stroke, vertebrobasilar flow, ROC
Zhang et al. [51]	MRI-confirmed medullary infarction	VAH/V4 hypoplasia	No VAH	Medullary infarction subtype, VAH prevalence, outcomes
Gilberti et al., [52]	Isolated midbrain infarction	VAH <2 mm	No control	Midbrain infarction, vertebrobasilar hypoplasia

Supplementary Table S4. Quality assessment of studies investigating VAH and posterior circulation ischemia

Study	Q1 Inclusion criteria	Q2 Study subjects	Q3 Exposure measured	Q4 Standard criteria	Q5 Confounders identified	Q6 Confounding addressed	Q7 Outcomes measured	Q8 Statistical analysis	Overall RoB
Hu et al. [36]	Yes	Yes	Yes	Yes	Yes	Yes	Yes	Yes	Low
Yang et al [37]	Yes	Yes	Yes	Yes	Partial	Partial	Yes	Yes	Moderate
Perren et al. [38]	Yes	Yes	Yes	Yes	Partial	Partial	Yes	Yes	Moderate
Kulyk et al. [39]	Yes	Yes	Yes	Yes	Yes	Yes	Yes	Yes	Low
Gaigalaite et al. [40]	Yes	Yes	Yes	Yes	Yes	Yes	Yes	Yes	Low
Gaigalaite et al. [41]	Yes	Yes	Yes	Yes	Yes	Yes	Yes	Yes	Low
Li et al. [42]	Yes	Yes	Yes	Yes	Yes	Yes	Yes	Yes	Low
Vilimas et al. [34]	Yes	Yes	Yes	Yes	Yes	Yes	Yes	Yes	Low
Zhang et al. [43]	Yes	Yes	Yes	Yes	Yes	Yes	Yes	Yes	Low
Zhang et al. [44]	Yes	Yes	Yes	Yes	Yes	Yes	Yes	Yes	Low
Chuang et al [45]	Yes	Yes	Yes	Yes	Yes	Yes	Yes	Yes	Low
Park et al. [46]	Yes	Yes	Yes	Yes	Partial	Partial	Yes	Yes	Moderate
Chuang et al., [47]	Yes	Yes	Yes	Yes	Yes	Yes	Yes	Yes	Low
Dinç et al., [48]	Yes	Yes	Yes	Yes	Yes	Yes	Yes	Yes	Low
Bakalarz et al., [49]	Yes	Yes	Yes	Yes	Partial	No	Yes	Yes	Moderate
Chi et al. [50]	Yes	Yes	Yes	Yes	Yes	Yes	Yes	Yes	Low
Zhang et al. [51]	Yes	Yes	Yes	Yes	Yes	Yes	Yes	Yes	Low
Gilberti et al., [52]	Yes	Yes	Yes	Yes	Partial	No	Yes	Partial	Moderate

Supplementary Table S5. Characteristics of studies assessing hemodynamic alterations associated with vertebral artery hypoplasia

Study	Population (P)	Exposure (E)	Comparison (C)	Outcomes (O)
Sato et al. [53]	Healthy young women	VAH / vertebral artery insufficiency	Controls; VAH vs VAI	Blood flow, CO_2 reactivity, cerebrovascular regulation
Chen et al [31]	Health-screening population	VAH (Doppler US)	Non-VAH	Flow volume, flow asymmetry, low-flow prevalence
Wang et al. [33]	Symptomatic patients	VAH / vertebral artery stenosis	VAH vs stenosis; affected vs unaffected side	PSV, EDV, RI, diagnostic accuracy
Chi et al. [54]	Stroke patients	VAH and/or VBD	No VAH/VBD; VAH; VBD	Flow volume, flow ratio, posterior stroke
Bao et al., [55]	Patients with spontaneous VAD	VAH	Contralateral unaffected VA	CFD hemodynamic parameters (TAWSS, OSI, ECAP, RRT)
Zarrintan et al. [56]	Adults with and without VAH	VAH	Healthy controls	PSV, RI, vessel diameter
Mitsumura et al. [57]	Acute ischemic stroke patients	VAH	Non-VAH	VA occlusion, infarct localization
Szárazová et al. [58]	Posterior circulation stroke	VAH	Non-VAH	Stroke characteristics, recurrence, risk factors
Hsu et al. [59]	Acute ischemic stroke patients	Unilateral/bilateral VAH	No VAH	Infarct pattern, vertebral flow, fetal PCA
Chi et al. [50] .	AIS patients (posterior vs anterior circulation)	VAH (≤2.2 mm) with Doppler flow abnormalities	Posterior vs anterior stroke; VAH vs non-VAH	Flow volume, vertebrobasilar hemodynamics, ROC analysis, posterior stroke

Supplementary Table S6. Quality assessment of studies evaluating the hemodynamic consequences of VAH

Study	Q1 Inclusion criteria	Q2 Study subjects	Q3 Exposure measured	Q4 Standard criteria	Q5 Confounders identified	Q6 Confounding addressed	Q7 Outcomes measured	Q8 Statistical analysis	Overall RoB
Sato et al. [53]	Yes	Yes	Yes	Yes	Yes	Yes	Yes	Yes	Low
Chen et al [31]	Yes	Yes	Yes	Yes	Partial	No	Yes	Yes	Moderate
Wang et al. [33]	Yes	Yes	Yes	Yes	Partial	Partial	Yes	Yes	Moderate
Chi et al. [54]	Yes	Yes	Yes	Yes	Partial	Partial	Yes	Yes	Moderate
Bao et al., [55]	Yes	Yes	Yes	Yes	Partial	Partial	Yes	Yes	Moderate
Zarrintan et al. [56]	Yes	Yes	Yes	Yes	Partial	Partial	Yes	Yes	Moderate
Mitsumura et al. [57]	Yes	Yes	Yes	Yes	Partial	Yes	Yes	Yes	Low
Szárazová et al. [58]	Yes	Yes	Yes	Yes	Yes	Partial	Yes	Yes	Moderate
Hsu et al. [59]	Yes	Yes	Yes	Yes	Partial	No	Yes	Partial	Moderate
Chi et al. [50]	Yes	Yes	Yes	Yes	Yes	Yes	Yes	Yes	Low

Supplementary Table S7. Characteristics of studies investigating clinical manifestations associated with vertebral artery hypoplasia

Study	Population (P)	Exposure (E)	Comparison (C)	Outcomes (O)
Werner et al. [60]	Neurological patients (TGA cohort + controls)	VAH (TOF-MRA)	Controls without VAH	Hemodynamics, VA diameter
Zhang et al. (vertigo cohort) [43]	Vertigo patients	VAH (MRA/CEMRA)	Non-VAH	Posterior circulation infarction
Pašaoglu et al., [61]	Central vertigo patients	VAH / VA stenosis	Controls	Vertebrobasilar abnormalities
Chuang et al., [45]	Vestibular neuritis patients	Intracranial VAH	Healthy controls	VAH prevalence, vascular risk
Gaigalaite et al., [40]	PCS/TIA patients + controls	VAH (<3 mm)	Asymptomatic controls	PCS/TIA risk
Szárazová et al. [58]	Posterior stroke patients	VAH	Non-VAH	Stroke localization
Giannopoulos et al., [62]	Young patients with vertebrobasilar ischemia	VAH	Contralateral VA	Lateral medullary infarction
Luan et al. [63]	Single patient	VA stenosis + VAH	Pre/post intervention	Symptom improvement
Cai et al. [64]	Single patient (Bow Hunter syndrome)	VAH + dynamic compression	Pre/post-surgery	Vertebrobasilar insufficiency
Bae et al., [65]	Cardioembolic stroke patients	Left VAH	Non-VAH	BAO, functional outcome
Chung & Sovory [66]	Single patient	VAH	No formal comparator	Lateral medullary infarction
Çiçek et al., [29]	Patients with headache, neck pain and vertigo	VAH + arcuate foramen	Without VAH / without AF	Vertigo, anatomical variants
Vynichakis et al. [67]	Single trauma patient	VAH	No comparator	Post-traumatic vertigo, vertebrobasilar insufficiency
Mestan [68]	Single patient with cerebellar infarction	Bilateral VAH	No comparator	Posterior fossa ischemia
Jianu et al., [69]	Single patient with pontine infarction	VAH + congenital vascular anomalies	No comparator	Brainstem ischemia
Ueki et al. [70]	Single patient with embolic stroke	VAH + protein C deficiency	No comparator	Recurrent posterior circulation embolism

Supplementary Table S8. Quality assessment of studies evaluating clinical manifestations of VAH

Study	Q1 Inclusion criteria	Q2 Study subjects	Q3 Exposure measured	Q4 Standard criteria	Q5 Confounders identified	Q6 Confounding addressed	Q7 Outcomes measured	Q8 Statistical analysis	Overall RoB
Werner et al. [60]	Yes	Yes	Yes	Yes	Yes	Yes	Yes	Yes	Low
Zhang et al. (vertigo cohort) [43]	Yes	Yes	Yes	Yes	Yes	Yes	Yes	Yes	Low
Pašaoglu et al., [61]	Yes	Yes	Yes	Yes	Partial	Partial	Yes	Yes	Moderate
Chuang et al., [45]	Yes	Yes	Yes	Yes	Yes	Yes	Yes	Yes	Low
Gaigalaite et al., [40]	Yes	Yes	Yes	Yes	Yes	Yes	Yes	Yes	Low
Szárazová et al. [58]	Yes	Yes	Yes	Yes	No	Partial	Yes	Yes	Moderate
Giannopoulos et al., [62]	Partial	Yes	Yes	Yes	Partial	No	Yes	No	High
Luan et al. [63]	No	Yes	Yes	Yes	Partial	No	Yes	No	High
Cai et al. [64]	Partial	Yes	Yes	Yes	No	No	Yes	No	High
Bae et al., [65]	Yes	Yes	Yes	Yes	Partial	Yes	Yes	Yes	Low
Chung & Sovory [6]	No	Yes	Yes	Yes	No	No	Yes	No	High
Çiçek et al., [29]	Yes	Yes	Yes	Yes	No	No	Yes	Yes	Mode
Vynichakis et al. [67]	No	Yes	Yes	Yes	Partial	Partial	Yes	No	High
Mestan [68]	No	Yes	Yes	Yes	No	No	Yes	No	High
Jianu et al., [69]	No	Yes	Yes	Yes	Partial	No	Yes	No	High
Ueki et al. [70]	No	Yes	Yes	Yes	Partial	Yes	Yes	No	High

Supplementary Table S9. Characteristics of studies assessing anatomical and vascular variations associated with vertebral artery hypoplasia

Study	Population (P)	Exposure (E)	Comparison (C)	Outcomes (O)
Li et al. [42]	TIA, stroke patients + controls	VAH (MRA)	Healthy controls / non-VAH	TIA, infarction, stenosis
Gaigalaite et al., [41]	PCS/TIA patients + controls	VAH + CoW variants	Non-VAH	PCS/TIA risk
Fortuniak et al. [27]	AIS patients	VAH (US criteria)	Non-VAH	Posterior stroke, RI
Rajasekhar et al. [71]	Cadaveric case	VAH with vascular anomalies	Normal anatomy	Anatomical variation
Layegh et al. [72]	Posterior stroke patients	VAH	Non-VAH	Infarct distribution
Chi et al. [54]	Stroke patients	VAH + VBD	Non-VAH/VBD	Posterior stroke, flow volume
Siva et al. [32]	CTA patients	VAH and VA variants	Normal VA	Anatomical variability

Supplementary Table S10. Quality assessment of studies evaluating anatomical and vascular characteristics associated with VAH

Study	Q1 Inclusion criteria	Q2 Study subjects	Q3 Exposure measured	Q4 Standard criteria	Q5 Confounders identified	Q6 Confounding addressed	Q7 Outcomes measured	Q8 Statistical analysis	Overall RoB
Li et al. [42]	Yes	Yes	Yes	Yes	Partial	No	Yes	Yes	Moderate
Gaigalaite et al., [41]	Yes	Yes	Yes	Yes	Yes	Yes	Yes	Yes	Low
Fortuniak et al. [27]	Yes	Yes	Yes	Yes	Yes	Partial	Yes	Yes	Low–
Rajasekhar et al. [71]	Yes	Yes	Yes	Yes	No	No	Yes	No	High
Layegh et al. [72]	Yes	Yes	Yes	Yes	Partial	No	Yes	Yes	Moderate
Chi et al. [54]	Yes	Yes	Yes	Yes	Partial	Partial	Yes	Yes	Moderate
Siva et al., [32]	Yes	Yes	Yes	Yes	Partial	Partial	Yes	Yes	Moderate

Supplementary Table S11. PRISMA 2020 Checklist

Section and Topic	Item #	Checklist item	Location where item is reported
**TITLE**			
Title	1	Identify the report as a systematic review.	Line 119
**ABSTRACT**			
Abstract	2	See the PRISMA 2020 for Abstracts checklist.	Line 26
**INTRODUCTION**			
Rationale	3	Describe the rationale for the review in the context of existing knowledge.	Line 105
Objectives	4	Provide an explicit statement of the objective(s) or question(s) the review addresses.	Line 112, 130
**METHODS**			
Eligibility criteria	5	Specify the inclusion and exclusion criteria for the review and how studies were grouped for the syntheses.	Line 150-183
Information sources	6	Specify all databases, registers, websites, organisations, reference lists and other sources searched or consulted to identify studies. Specify the date when each source was last searched or consulted.	Line 185
Search strategy	7	Present the full search strategies for all databases, registers and websites, including any filters and limits used.	Line 197
Selection process	8	Specify the methods used to decide whether a study met the inclusion criteria of the review, including how many reviewers screened each	Line 215

Section and Topic	Item #	Checklist item	Location where item is reported
		record and each report retrieved, whether they worked independently, and if applicable, details of automation tools used in the process.	
Data collection process	9	Specify the methods used to collect data from reports, including how many reviewers collected data from each report, whether they worked independently, any processes for obtaining or confirming data from study investigators, and if applicable, details of automation tools used in the process.	Line 210
Data items	10a	List and define all outcomes for which data were sought. Specify whether all results that were compatible with each outcome domain in each study were sought (e.g. for all measures, time points, analyses), and if not, the methods used to decide which results to collect.	Line 275
	10b	List and define all other variables for which data were sought (e.g. participant and intervention characteristics, funding sources). Describe any assumptions made about any missing or unclear information.	Line 257
Study risk of bias assessment	11	Specify the methods used to assess risk of bias in the included studies, including details of the tool(s) used, how many reviewers assessed each study and whether they worked independently, and if applicable, details of automation tools used in the process.	Line 286
Effect measures	12	Specify for each outcome the effect measure(s) (e.g. risk ratio, mean difference) used in the synthesis or presentation of results.	Line 328
Synthesis methods	13a	Describe the processes used to decide which studies were eligible for each synthesis (e.g. tabulating the study intervention characteristics and comparing against the planned groups for each synthesis (item #5)).	Line 337
	13b	Describe any methods required to prepare the data for presentation or synthesis, such as handling of missing summary statistics, or data conversions.	Line 349
	13c	Describe any methods used to tabulate or visually display results of individual studies and syntheses.	Line 354
	13d	Describe any methods used to synthesize results and provide a rationale for the choice(s). If meta-analysis was performed, describe the model(s), method(s) to identify the presence and extent of statistical heterogeneity, and software package(s) used.	NA
	13e	Describe any methods used to explore possible causes of heterogeneity among study results (e.g. subgroup analysis, meta-regression).	NA
	13f	Describe any sensitivity analyses conducted to assess robustness of the synthesized results.	NA
Reporting bias assessment	14	Describe any methods used to assess risk of bias due to missing results in a synthesis (arising from reporting biases).	Line 286
Certainty assessment	15	Describe any methods used to assess certainty (or confidence) in the body of evidence for an outcome.	Line 301
**RESULTS**			
Study selection	16a	Describe the results of the search and selection process, from the number of records identified in the search to the number of studies included in the review, ideally using a flow diagram.	Line 222
	16b	Cite studies that might appear to meet the inclusion criteria, but which were excluded, and explain why they were excluded.	/
Study characteristics	17	Cite each included study and present its characteristics.	Lines 397, 442, 485, 536, 580

Section and Topic	Item #	Checklist item	Location where item is reported
Risk of bias in studies	18	Present assessments of risk of bias for each included study.	Lines 361, 406, 451, 496, 546
Results of individual studies	19	For all outcomes, present, for each study: (a) summary statistics for each group (where appropriate) and (b) an effect estimate and its precision (e.g. confidence/credible interval), ideally using structured tables or plots.	Lines 397, 442, 485, 536, 580
Results of syntheses	20a	For each synthesis, briefly summarise the characteristics and risk of bias among contributing studies.	Lines 361, 406, 451, 496, 546
	20b	Present results of all statistical syntheses conducted. If meta-analysis was done, present for each the summary estimate and its precision (e.g. confidence/credible interval) and measures of statistical heterogeneity. If comparing groups, describe the direction of the effect.	NA
	20c	Present results of all investigations of possible causes of heterogeneity among study results.	NA
	20d	Present results of all sensitivity analyses conducted to assess the robustness of the synthesized results.	NA
Reporting biases	21	Present assessments of risk of bias due to missing results (arising from reporting biases) for each synthesis assessed.	Lines 361, 406, 451, 496, 546
Certainty of evidence	22	Present assessments of certainty (or confidence) in the body of evidence for each outcome assessed.	Lines 397, 442, 485, 536, 580
**DISCUSSION**			
Discussion	23a	Provide a general interpretation of the results in the context of other evidence.	Line 583
	23b	Discuss any limitations of the evidence included in the review.	Line 696
	23c	Discuss any limitations of the review processes used.	Line 696
	23d	Discuss implications of the results for practice, policy, and future research.	Line 708
**OTHER INFORMATION**			
Registration and protocol	24a	Provide registration information for the review, including register name and registration number, or state that the review was not registered.	Line 123
	24b	Indicate where the review protocol can be accessed, or state that a protocol was not prepared.	Line 123
	24c	Describe and explain any amendments to information provided at registration or in the protocol.	Line 123
Support	25	Describe sources of financial or non-financial support for the review, and the role of the funders or sponsors in the review.	Line 736
Competing	26	Declare any competing interests of review authors.	Line 735

Section and Topic	Item #	Checklist item	Location where item is reported
interests			
Availability of data, code and other materials	27	Report which of the following are publicly available and where they can be found: template data collection forms; data extracted from included studies; data used for all analyses; analytic code; any other materials used in the review.	Line 740
